# Supplementary material for: Multiple Modulation of Acid-Sensing Ion Channel 1a by the Alkaloid Daurisoline
Source: Biomolecules. 2019 Aug 2;9(8):336. doi: 10.3390/biom9080336 (PMC6722837; doi:10.3390/biom9080336)
Supplement: Supplementary file 1 [file biomolecules-09-00336-s001.pdf]

Supplementary Materials

We simulated the kinetic of proton-activated currents at the ASIC1a channel (taking into account the rate of change of the solution) using the method of numerical solution of a system of kinetic equations using an algorithm similar to that described [1]. The accuracy of this modeling method was verified by comparing our calculations with analytical solutions for a three-state [2] and four-state [3] models of receptor activation by the ligand. Comparison of dose-response curves fitted to the maximum amplitude of the current generated by our program with these analytical solutions showed complete coincidence of numerical simulation predictions (the relative amplitude error was less than 10^{-7}% over the entire range of ligand concentrations (10^{-9}–10^{-3} M). Thus, our algorithm can be successfully used to simulate the currents of ligand-activated receptors, including rapidly activated, such as ASIC channels.

As is known from the literature the activation of ASICs may occur after the binding of numerous protons (≤16) [4,5]. At first, to simulate the proton dependence of ASIC1a activation, we developed a theoretical Model 1 describing several consecutive closed states, which can eventually turn into an open or desensitized state:

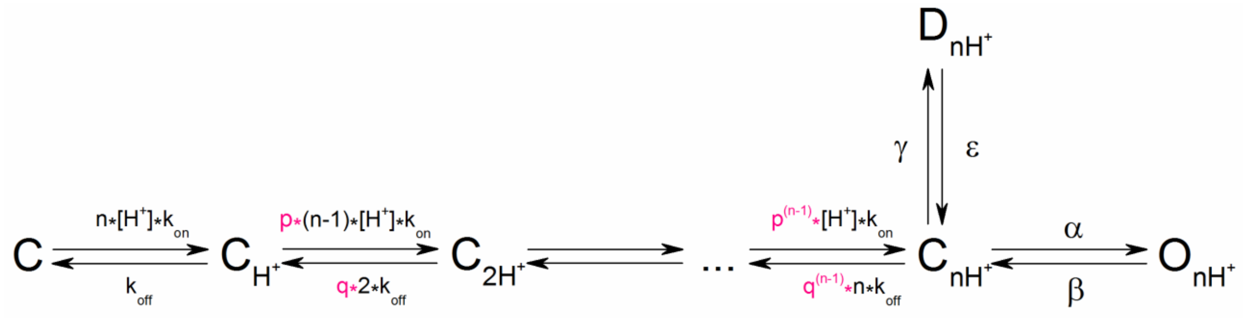

Scheme S1. Model 1/2. Model 2 with introduced cooperativity factors (p and q < 1).

The kinetic constants $k_{on} = 2 \times 10^9\ M^{-1}s^{-1}$ and $k_{off} = 10^3\ s^{-1}$ for protons binding to ASIC1a were suggested early in literature by [6]. Other constant: The rate constant of the channel opening $\alpha = 1000\ s^{-1}$; the rate constant of the channel closing $\beta = 1000\ s^{-1}$; the rate constants of the channel transition to desensitization $\gamma = 10\ s^{-1}$, and the rate constants of the channel transition from desensitization $\varepsilon = 0.02\ s^{-1}$, were chosen on the basis of the speed and magnitude of the desensitization current decrease. The coefficients "p" and "q", highlighted in red, reflect the cooperativity of the interaction of protons with the channel and they were introduced into the Model 2 (see description below).

We investigated dose-dependence of the probability of the channel opening for Model 1 with 3, 6, and 9 identical binding sites (M1-3, M1-6, M1-9). This pool of sites number was chosen in view of homotrimeric ASIC1a organization. All three models reproduced similar shape of the current generated by various proton

concentrations from standard conditioning pH 7.4 (change solution time is 50 ms; time application is 1 s) (Figure S1A).

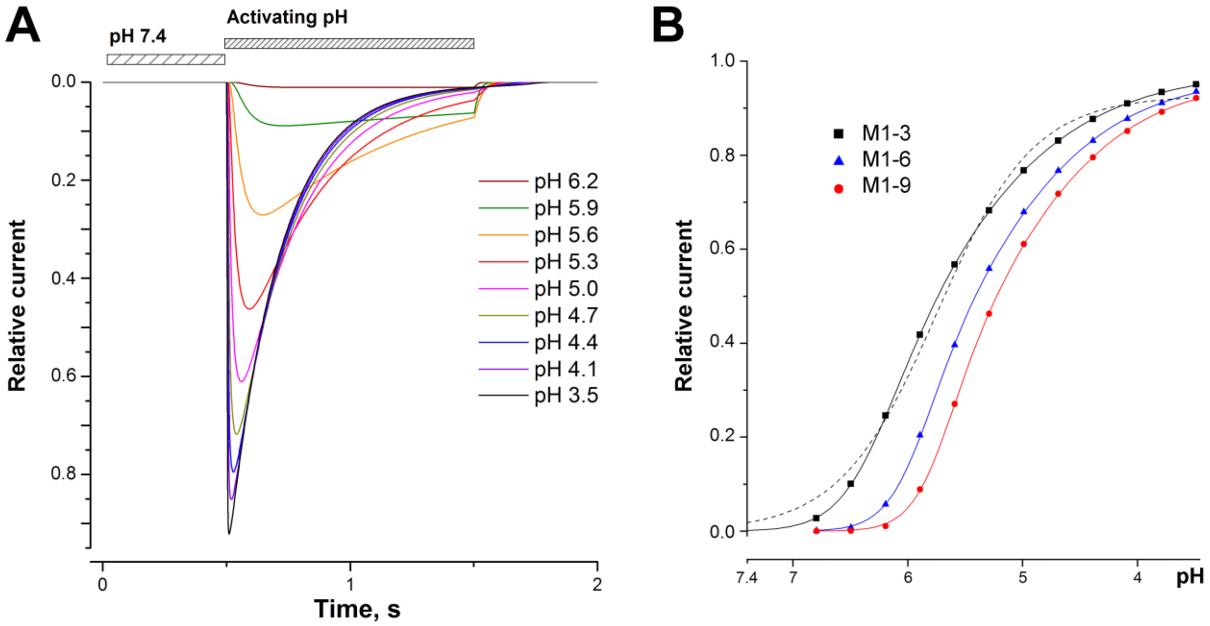

Figure S1. (**A**) The set of generated traces predicted for a model M1-9. (**B**) The dose dependence curves obtained for models M1-3, M1-6, M1-9 by fitting with equation F_2 (solid lines) and by logistic equation F_1 for model M1-3 (dash line).

The pH-dependence of the channel activation was analyzed for all three models (Figure S1B). In all cases the dependences of the maximum amplitude of the current versus proton concentration keep an asymmetrical shape of curve like the experimental curve, and poorly fitted by the logistic equation. Otherwise a reliable fitting of we fitted the dose-response with the following equation F_2:

$$F(x) = A/(1 + (x/[pH_{50}1])^{nH1}) \times (1 + (x/[pH_{50}2]^{nH2})).$$

The dose-dependence activation of all models is perfectly fitted by this equation. The Hill coefficients obtained by the fittings are shown in Table S1.

Table S1. The Hill coefficients trend for selected models.

	M1-3	M1-6	M1-9
n_H1	1.71	2.17	2.35
n_H2	0.61	0.68	0.70

The value of the n_H2 always remains less than one (as in the experimental data). The value n_H1, which determines the steepness of the left part of the proton-dependence curve of activation (Figure S1B) has a tendency to grow when a number of proton sites in Model 1 is increased, but this tendency is not enough to achieve higher n_H1 values. Therefore, the Model 1 without modification failed to obtain high n_H1 values close to experimental (more than 6) by a simple increasing of proton binding sites number.

In order to obtain higher n_H1, we designed the Model 2 with two parametric coefficients p and q. We assumed that the binding of protons is a cooperative

process, causing the conformational rearrangements in the channel that were described [7] as a collapse of the pocket where protons bind. As a result, each successive proton will have different binding/dissociation characteristics than the previous one. Thus, we believed that the rate constants of both binding and dissociation decrease with each subsequent process of interaction of the proton with the channel and introduced the cooperativity coefficient p < 1 (for binding constants) and coefficient q (for dissociation constants) that always less than p.

As a result, we managed to achieve an increase in the value of n_H1 (Figure S2A, B). For the M2-6, n_H1 does not exceed a value of 4.5 for any values of the "p" and "q" coefficients (Figure S2A). For the M2-9 model, the value of n_H1, about 6.4, is achieved only in a narrow range of q and p values. For further modeling, we take the q p and values of the main maximum (Figure S2B,C). Thus, we assume that the number of proton-binding sites equal to nine is a necessary and sufficient, and best calculation is produced for q coefficient of 0.65 and p coefficient of 0.75 (Figure S2D).

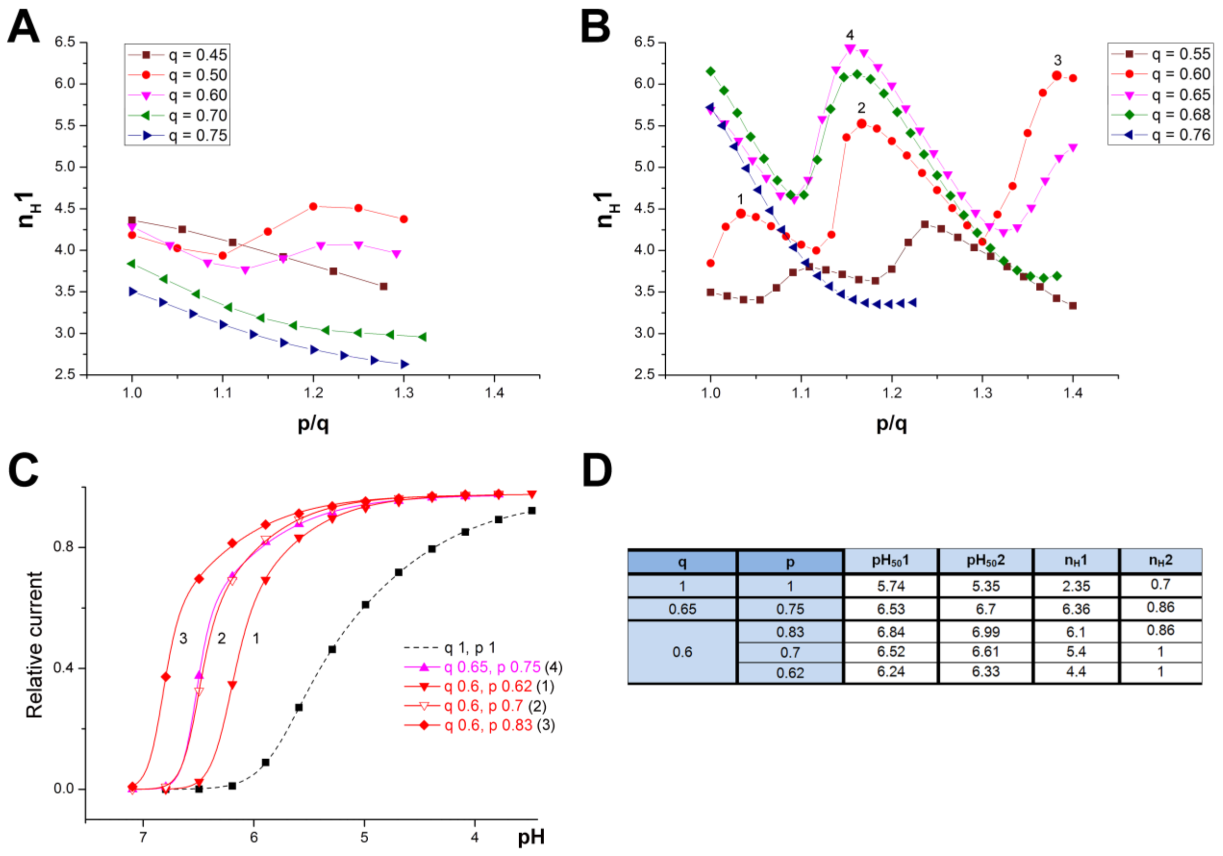

q	p	$pH_{50}1$	$pH_{50}2$	n_H1	n_H2
1	1	5.74	5.35	2.35	0.7
0.65	0.75	6.53	6.7	6.36	0.86
0.6	0.83	6.84	6.99	6.1	0.86
	0.7	6.52	6.61	5.4	1
	0.62	6.24	6.33	4.4	1

Figure S2. Optimization of the models. (AB) of the Plot of different variants of the q and p coefficients affecting the n_H1 coefficient in the model M2-6—panel (**A**) and model M2-9—panel (**B**). Dose-dependence curves (**C**) and tabular data (**D**) obtained by fitting with equation F_2 for M2-9 model with various cooperativity coefficients q and p (points 1–4 from Panel B) vs. control q = p =1.

It should be noted, that the M2-9 model with selected p and q values 0.75 and 0.65, respectively, give the satisfactory result for n_H1 and n_H2 but false $pH_{50}1$ and $pH_{50}2$ values. Experimental $pH_{50}1$ is greater than $pH_{50}2$ (Figure 6a in main text) while this model produce more high value for $pH_{50}2$ (Figure S2D). To overcome this inconsistency, we introduced into Model 2 an additional proton binding site with and noncooperative less affine site (Model 3; see also the scheme and text in the main results).

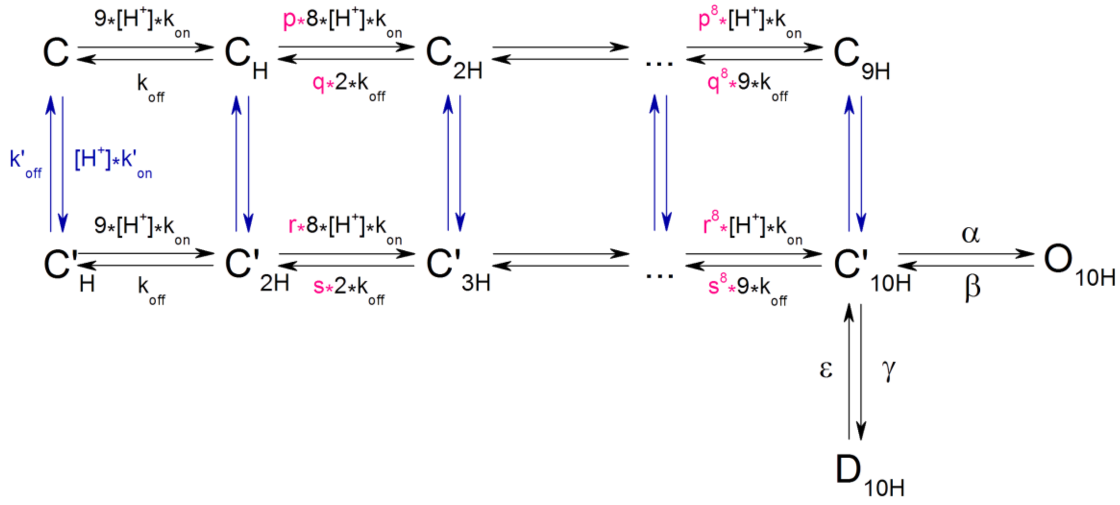

Scheme S2. Model 3

We introduced the following assumptions into this model:

1) The tenth site is low affine, $k'_{on} = 2 \times 10^9\ M^{-1}s^{-1}$ and $k'_{off} = 1.2 \times 10^3\ s^{-1}$ (the last constant was taken based on the difference between $pH_{50}1$ and $pH_{50}2$ in the experiment);

2) Proton binding in the main (highly cooperative) pool does not affect proton binding in the low affinity region;

3) Proton binding in the low affinity site also does not affect proton binding in the cooperative pool, i.e., p = r, q = s.

Under these assumptions, the entire dose-dependence is shifted to the right in the region of acidic pH. Returning the curve to the left is possible by increasing the cooperativity of the binding by increasing the coefficients of cooperativity p and r. This allowed us to obtain a dose-dependence, which is fitted by equation F_2 with parameter values close to the experimental ones (see Table S2).

Table S2. Comparison of the parameters of the pH dependence of the model M3-10 activation with non-fixed and fixed per unit coefficient n_H2.

	$pH_{50}1$	$pH_{50}2$	n_H1	n_H2
**M3-10 (n_H2 free)**	6.62	6.61	5.6	0.77
**M3-10 (n_H2 fixed)**	6.65	6.53	6.4	1
**Oocyte experimental data**	6.67 ± 0.01	6.59 ± 0.01	6.7 ± 0.5	0.96 ± 0.06

It should be noted that, although the parameters of the fitting of the model agree well with the experimental data, the coefficient n_H1 becomes smaller. This can be compensated for by increasing the number of highly operational sites, or by fixing the second coefficient when fitted by equation F_2. Since the experimental data showed the second coefficient very close to 1, we adjusted the data of the M3-10 model with the coefficient n_H2 fixed to the value 1. These fittings are shown in the main text and in Figure 6.

References

1. Benveniste, M.; Clements, J.; Vyklický, L.; Mayer, M.L. A kinetic analysis of the modulation of N-methyl-D-aspartic acid receptors by glycine in mouse cultured hippocampal neurones. *J. Physiol.* **1990**, *428*, 333–357, doi:10.1113/jphysiol.1990.sp018215.
2. Colquhoun, D.; Hawkes, A.G. Relaxation and fluctuations of membrane currents that flow through drug-operated channels. *Proc. R. Soc. London. Ser. B. Biol. Sci.* **1977**, *199*, 231–262, doi:10.1098/rspb.1977.0137.
3. Patneau, D.K.; Mayer, M.L. Structure-activity relationships for amino acid transmitter candidates acting at N-methyl-D-aspartate and quisqualate receptors. *J. Neurosci.* **1990**, *10*, 2385–99, doi: 10.1523/JNEUROSCI.10-07-02385.1990.
4. Ishikita, H. Proton-Binding Sites of Acid-Sensing Ion Channel 1. *PLoS One* **2011**, *6*, e16920, doi:10.1371/journal.pone.0016920.
5. Vullo, S.; Bonifacio, G.; Roy, S.; Johner, N.; Bernèche, S.; Kellenberger, S. Conformational dynamics and role of the acidic pocket in ASIC pH-dependent gating. *Proc. Natl. Acad. Sci.* **2017**, *114*, 3768–3773, doi:10.1073/pnas.1620560114.
6. Gründer, S.; Pusch, M. Biophysical properties of acid-sensing ion channels (ASICs). *Neuropharmacology* **2015**, *94*, 9–18, doi:10.1016/j.neuropharm.2014.12.016.
7. Yoder, N.; Yoshioka, C.; Gouaux, E. Gating mechanisms of acid-sensing ion channels. *Nature* **2018**, *555*, 397–401, doi:10.1038/nature25782.
